# A computational model for driver's cognitive state, visual perception and intermittent attention in a distracted car following task

**DOI:** 10.1098/rsos.180194

**Published:** 2018-09-05

**Authors:** Jami Pekkanen, Otto Lappi, Paavo Rinkkala, Samuel Tuhkanen, Roosa Frantsi, Heikki Summala

**Affiliations:** 1 Cognitive Science, PO Box 9, 00014 University of Helsinki, Finland; 2 TRUlab, Department of Digital Humanities, PO Box 9, 00014 University of Helsinki, Finland; 3 Helsinki Center for Digital Humanities (HELDIG), Finland; 4 Spatial Planning and Transportation Engineering, Department of Built Environment, Aalto University, Finland

**Keywords:** visual attention, top-down control, natural task performance, driving, predictive processing, cognitive modelling

## Abstract

We present a computational model of intermittent visual sampling and locomotor control in a simple yet representative task of a car driver following another vehicle. The model has a number of features that take it beyond the current state of the art in modelling natural tasks, and driving in particular. First, unlike most control theoretical models in vision science and engineering—where control is directly based on observable (optical) variables—actions are based on a temporally enduring internal representation. Second, unlike the more sophisticated engineering driver models based on internal representations, our model explicitly aims to be psychologically plausible, in particular in modelling perceptual processes and their limitations. Third, unlike most psychological models, it is implemented as an actual simulation model capable of full task performance (visual sampling and longitudinal control). The model is developed and validated using a dataset from a simplified car-following experiment (*N* = 40, in both three-dimensional virtual reality and a real instrumented vehicle). The results replicate our previously reported connection between time headway and visual attention. The model reproduces this connection and predicts that it emerges from control of action uncertainty. Implications for traffic psychological models and future developments for psychologically plausible yet computationally rigorous models of full natural task performance are discussed.

## 1. Background

Much of human behaviour takes place in complex, uncertain, time-critical environments where errors carry undesirable, even potentially catastrophic, consequences. Everyday examples include cooking, childcare and traffic. The same is true of many professional activities such as piloting an aircraft and most forms of sport. The apparent ease with which skilled individuals function in such environments belies the sophistication required from the underlying cognitive and perceptual systems. These requirements become apparent when artificial intelligence and robotic solutions are to be designed. Tasks that proficient humans would deem almost trivial turn out to present formidable computational challenges—one prime example being autonomous vehicles in normal traffic.

It is thus timely and important for cognitive science to further our understanding of the underpinnings of our rather remarkable everyday performance. This can be achieved by experimentation and modelling of extended, natural tasks representative of the real world. Driving is one such domain, and in many ways an excellent model domain to study. It is a ubiquitous task in modern society, exemplifies many fundamental perceptual–motor and attentional processes, and representative tasks can be presented at various levels of complexity (e.g. from lane keeping on a straight road to negotiating busy traffic or a sequence of bends at speed). What is more, among real-world tasks it stands out in terms of the state of maturity of modelling: real chunks of human behaviour can be understood quantitatively, and computational models can be developed that can actually perform the same (sub)tasks as experiment participants. This is not the case for most experimentally studied real-world tasks (such as making tea or sandwiches).

Following another vehicle on a road with no intersections or junctions is perhaps the simplest, and therefore most understandable, common routine subtask in driving. It is also one of the most in-depth modelled forms of real-world human behaviour, having received decades of interest from traffic psychologists and vehicle/road engineers alike. Car following (CF) models have undeniable practical importance in engineering roads for more efficient traffic flow and safety. But additionally, for basic researchers interested in rigorous modelling of everyday behaviour, it also happens to be one of the most constrained and stereotypical tasks that major parts of the population regularly conduct. This characteristic, combined with the rich literature, makes CF a useful model environment for studying more general mechanisms behind driving and other locomotor behaviours. In this paper, we present a computational model of intermittent visual sampling and locomotor control in this simple yet representative natural task.

### 1.1. Car following models and human factors

A car driver following another vehicle has been a subject of mathematical and computational modelling for over half a century and the resulting CF models have been successful in capturing and explaining various driver and especially traffic level phenomena (for review, see [1]). These so-called microsimulation models typically model the driver as a stimulus–response system, where the driver reacts to an observed situation by adjusting their speed. The reactions are usually modelled as accelerations which are integrated over time to produce a trajectory of the vehicle.

A common criticism of these models has been that they overlook many known ‘human factors’ of driving behaviour [2–4]. Many of these factors—e.g. limited perception accuracy, significant and varying reaction times, imperfect control, fluctuations in attention and changes in motivation—have been addressed in various CF models, especially in relatively recent developments (for a review, see [5]). Some especially relevant work for the current article includes optically plausible perception models [6–10]; models including temporally enduring cognitive state, such as memory [11,12]; models including attentional mechanisms [8,13,14]; and stochastic formulations [6,8,15–17].

One commonality in most of these models is that they model the human factors in a somewhat ‘ad hoc’ manner, i.e. they generally alter some input or output subsystem be more in line with what is known about human psychophysiology, but do not base the behaviour on more general cognitive and psychological mechanisms. This is perfectly reasonable for *traffic engineering*, where the traffic-level phenomena arising from multiple drivers interacting is seen as the emergent effect and object of interest. But this is in contrast to much of traffic psychology, where the focus is on driver behaviour arising from underlying psychological mechanisms.

A notable exception are the CF models based on stochastic utility maximization, which frame the driving process as a deliberate balancing of benefits gained from progress of the journey versus potential disutility due to a crash, and are able to show that known driver-level and traffic-level phenomena can emerge from this process [15,16]. Utility maximization models are also of interest in connection to conceptual traffic psychological models, as they explicitly use the subjective estimate of risk, which has long been a contentious topic in traffic psychology.

### 1.2. Traffic psychological driver models

For the benefit of the development of integrative models, the traffic psychology literature offers a number of conceptual driver models. While not defined in rigorous mathematical formulations or implemented computationally, these can be valuable for inspiration and analysis of the driving (sub)tasks. The literature is rich and diverse, and we will here consider only the models that have most directly contributed to the development of the present work.

Zero Risk Theory (ZRT) of Näätänen & Summala [18,19] was one of the first well-developed psychological driver models and has been updated in a number of iterations [20–23]. Its key posit is that driving is most of the time an automatic, routine task of controlling vehicle speed and trajectory so as to maintain safety margins (such as lane position or time to line crossing, or following distance or time headway to leading vehicle). These are kept at a level which simultaneously satisfies a motive for making progress and remain in a subjective ‘comfort zone’. Importantly, to keep a sense of control, a driver maintains an awareness of the state of the driving situation, called expectancy (defined as ‘vivid, perception-like predictions’ [19, p. 188]), which is the basis for choosing control actions. As long as the expectancy is satisfied, there is no need to deviate from current routine action, and the situation is not experienced to involve subjective risk—hence the name Zero Risk Theory. Expectancy violations occur when safety margins cannot be maintained within the comfort zone—they are said to cross a risk monitor activation threshold—which is experienced as subjective risk, and calls for modifying ongoing behaviour (*viz*. choosing to maintain larger safety margins).

Another theoretical line of inquiry is the so-called ‘risk control models’ [24–26], which posit that the driving process emerges from continuously keeping subjectively estimated risk at some suitable, non-zero level. In these models, the driver is assumed to continuously compute an estimate of level of risk of a situation and act to bring it closer to the target level, e.g. speeding up when the estimated risk is too low or slowing down when it is too high. Much of the discussion has been directed to how different aspects, such as policy changes and safety equipment, affect the subjective risk. In the core however they do, more or less explicitly, assume that the cost of perceived risk is balanced against some perceived benefit, such as progress of the journey, to maximize some utility, which relates them to the previously discussed utility maximization CF models.

The task–capability interface (TCI) model sidesteps the issue of risk by formulating the driving process using demands of driving tasks and driver capabilities of conducting them [27]. Drivers operate to maintain a preferred level of task difficulty which is a difference between perceived task demand and capability. TCI has recently been given a computational expression to model effects of distraction in CF models [13,14,28]. Pekkanen *et al*. [28], further, used the occlusion method of Senders [29] (see also [30–32]) to investigate intermittency in visual sampling. A driver cannot be assumed to expend continuous attention to the driving task, but more likely only samples the visual scene ‘just in time’ and on a ‘need to know’ basis. This intermittency of attention can be considered as a (voluntary) reduction of driving capability to respond to a situation (such as a secondary task), which is compensated by a reduction in the task demand by increasing safety margins, so as to maintain a constant level of task difficulty. Mechanistically, and directly relevant to the present model, control during the occlusion has been considered to be driven, and more to the point sampling itself is likely to be driven by accumulation of uncertainty in an internal model maintained in short-term memory across samples. This type of internal model is posited in many theories, and slightly different versions are called expectancy [19], ‘image’ [29] or ‘visual buffer’ [33].

When driver behaviour is analysed (quantitatively) in the traffic psychology literature, this is usually done in terms of individual ‘perceptually available’ variables (e.g. time-to-contact, time-to-line-crossing), on which control is directly based. For example, empirical studies may seek *threshold values* of such variables where some psychological effect occurs or an action is initiated [11,34–36], desired values (*safety margins*) drivers habitually maintain [28,37–41] or *reaction times* to unexpected critical events [42–44].

A fundamental and well-known limitation of the approach of reducing complex behaviour into an aggregate parameter, and then investigating whether various independent variables (e.g. manipulated stimulus information, driver distraction, various human factors such as age, inebriation or drowsiness) have a statistical effect on this single dependent variable is that it fails to sufficiently constrain hypotheses to allow the development of mechanistic models [45,46].

The vehicle control and traffic simulation literature in engineering, on the other hand, provides computational process models (reviewed in [47–49]). But they incorporate little about the limitations of human sensory physiology and attention, or conversely, the power of memory and internal models [3,50]. Combining the approaches for mutual benefit therefore seems desirable.

### 1.3. Aims of the paper

In what follows, we make use of the formalisms of CF models, combined with findings and insights from the fields of traffic psychology, psychophysics and cognitive science to propose a quantitative computational model of driver's internal state during the task of routine CF. In contrast to most existing *engineering* models, we aim for psychological plausibility of the mechanisms underlying control. In contrast to most *psychological* models, we use a rigorous mathematical definition, and implement the model as a computational simulation.

Most CF models are based on the simplifying modelling assumption that in a single-lane road, the driver's speed control can be usefully approximated with a function of three *scene variables*: the driver's own speed, and the distance and relative speed to the leading vehicle [1]. Importantly, when the speed adjustments of such a function are integrated over time, the resulting dynamical system provides a mechanistic description of a driver's actions and can quantitatively simulate (very idealized) real-life human behaviour. Traditionally, these models are used in traffic engineering to study traffic-level phenomena emerging from interaction of such agents. In this work, we however use the CF setting to quantitatively model the underlying cognitive mechanisms in this ubiquitous real-life task. In addition to longitudinal control, we model a mechanism for intermittent visual attention during CF. Specifically, we model the driver's cognitive system as a stochastic internal model maintaining estimates of the relevant state variables, and uncertainty-driven top-down attentional processes that update these estimates through perceptual input modelled in a psychophysiologically motivated way.

Our main interest lies in control principles that are robust and flexible enough to generalize to other driving subtasks and beyond, but we use the simple task of CF as the test case for model development. The situation is already formally quite well understood, and can be effectively described in terms of a few key state variables, and parameter values to constrain model behaviour are available from the psychophysics and driver modelling literature.

Where the current model goes beyond most existing driver models is that it combines the following features into one model:

(i) Complete task performance—i.e. *the entire perceptual-motor loop*—is modelled: the model both controls the vehicle and performs active visual sampling to pick up the information.
(ii) To deal with intermittency in available visual information—inherent in natural driving behaviour [51–53]—control is based on a *stochastic internal representation* (of the key state variables), rather than on directly observed environment.
(iii) The internal representation is updated by *noisy perceptual inputs* that are based on a psychologically plausible perception model (rather than the environment's state being ‘directly available’ to the model).
(iv) *Visual sampling is driven by uncertainty* in the underlying internal representation (cf. [29,54]).

The perhaps most crucial novel aspect of our model is that attention is driven by *action* uncertainty—i.e. the need to be confident on *what action* to take—not *perceptual* or *environment state* uncertainty themselves. Technically, this is an elegant solution to the problem of relevance: if a control rule is available, it already includes the information of what is relevant for conducting the task. Conceptually, it embodies the idea that the task of perception is to pick up information that is relevant for action, not necessarily for inferring a complete and veridical ‘world model’.

For development, parametrization and empirical evaluation of the model we use data from a driving experiment where 40 participants drove simplified CF scenarios in both a real car (on a test track) and immersive virtual reality (VR) simulation. Intermittent allocation of overt attention was indexed with self-paced visual occlusion [28,29,32]. We earlier reported a relationship between time headway and occlusion duration in this setting [28], which we now replicate in more ecologically valid immersive three-dimensional VR and real driving tasks, and go further to provide a possible mechanistic explanation in terms of a fully implemented computational model. Limitations and future developments of the model to cover more varied driving scenarios and other forms of real-world behaviour are discussed.

## 2. Model description

**Figure 1.**
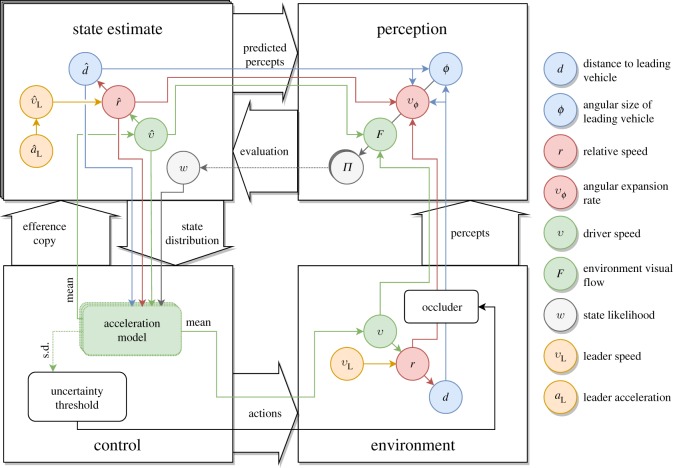
Schematic diagram of the proposed model. The large white boxes and arrows represent the components and their interactions on a conceptual level. A stochastic *state estimate* evolves according to predicted dynamics which are evaluated by the *perception model* against percepts induced by the *environment*. A distribution of desired accelerations is computed from the state distribution by the *control* model. Mean of the acceleration distribution is actuated to the vehicle and standard deviation measures action uncertainty, which controls the allocation of overt attention. The smaller coloured elements and arrows represent variables and their quantitative interactions. See the main text for details.

**Figure 2.**
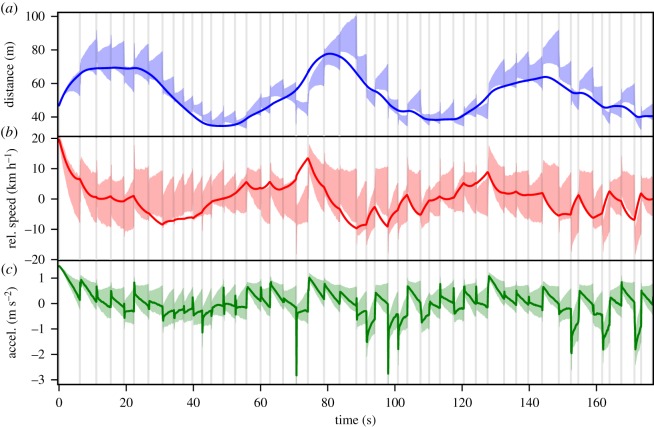
Time-evolution of three of the model's internal scene variable estimates when driving behind a vehicle with a sinusoidal speed profile. Solid lines display the true values of the state variables. The shaded area gives 25th to 75th percentile range of the internal stochastic estimate of the variables. Grey vertical lines indicate moments when the model requests a visual sample of the leading vehicle by removing the occlusion (see §2.3). The relative speed (*a*, blue) and distance (*b*, red) variables estimate the environment value. The acceleration variable (*c*, green) represents the *desired* acceleration of the driver based on the environment state estimate. Note that for simplicity the model can directly control the acceleration (desired acceleration is equal to the true acceleration), and thus can produce physically unrealistic values and variation (see §5.4 for further discussion).

The central assumption in our model is that—from the cognitive perspective—actions and perceptions are based on an internal *state representation*, which keeps a stochastic estimate of the relevant environmental state for the given task. The state representation is governed by prior assumptions about both the environment's dynamics and percepts induced by the environment. For an overview, the model's main components and their interactions are illustrated in figure 1, and a concrete example of evolution of the internal state is shown in figure 2. The following sections describe the model first from a conceptual, psychological perspective and each section concludes with a formal mathematical presentation. Mathematically, the state estimation model is a sequential Bayesian filter (e.g. [55]), and its parametrization and computational implementation as a particle filter are presented in §3.3.

### 2.1. State estimate and prediction

In the task of CF, we follow the usual assumption that at a given time instant *t*^1^ the relevant information about the environment is reasonably well captured by three *state variables*: the driver's own speed *v*[*t*], distance to the leading vehicle *d*[*t*] and speed relative to the leading vehicle *r*[*t*]. The driver's stochastic estimates of these variables form the *state estimate*, which is modelled as a random vector $\hat{S}[t]≜(\hat{v}[t],\hat{d}[t],\hat{r}[t])$.

We assume for simplicity that the driver can do various transformations of and between these variables exactly. More transformations are described in further sections, but importantly for the state prediction, the transformations between relative speed and leader speed, e.g. ${\hat{v}}_{L}[t]=\hat{v}[t]+\hat{r}[t]$, are assumed to be available.

The driver has also a stochastic model of some of the environment's dynamics, namely predicted leader acceleration ${\hat{a}}_{L}$ and an ‘efference copy’ $\bar{a}[t-\Delta t]$ of the own desired acceleration at the previous time instant (see §2.3), which the driver considers a noisy version of the actuated acceleration. The driver's and leader's accelerations are used to update the estimate of the driver's and leader's speeds. The relative speed is integrated to update the distance estimate. For simplicity, the integration is assumed to be done exactly. Importantly for modelling intermittent visual sampling, this produces state estimates even without sensory information from the environment.

The leading vehicle's acceleration is predicted to be normally distributed: ${\hat{a}}_{L}∼N(0,\sigma_{a_{L}}^{2})$. Own acceleration is predicted as the efference copy acceleration, but corrupted in the actuation process by normally distributed noise with standard deviation related to the acceleration's magnitude:^2^$\hat{a}∼N(\bar{a},{\left(\lambda_{a}\bar{a}\right)}^{2})$. These are used to form the *predicted state* ${\hat{S}}^{\prime }[t]$ at time *t*:^3^

2.1 $${\hat{S}}^{\prime }[t]≜({\hat{v}}^{\prime }[t],{\hat{d}}^{\prime }[t],{\hat{r}}^{\prime }[t]),$$

2.2 $${\hat{d}}^{\prime }[t]=\hat{d}[t-\Delta t]+\hat{r}[t-\Delta t]\Delta t,$$

2.3 $${\hat{v}}^{\prime }[t]=\hat{v}[t-\Delta t]+\hat{a}[t]\Delta t,\;\hat{a}[t]∼N(\bar{a}[t-\Delta t],{\left(\lambda_{a}\bar{a}[t-\Delta t]\right)}^{2}),$$

2.4 $${\hat{v}}_{L}^{\prime }[t]={\hat{v}}_{L}[t-\Delta t]+{\hat{a}}_{L}[t]\Delta t,\;{\hat{a}}_{L}[t]∼N(0,\sigma_{a_{L}}^{2})$$

and

2.5 $${\hat{r}}^{\prime }[t]={\hat{v}}_{L}^{\prime }[t]-{\hat{v}}^{\prime }[t].$$

### 2.2. Perception

After the new state distribution is predicted based on the dynamics, the prediction is ‘evaluated’ against percepts. The evaluation can be seen as giving more weight to the states that ‘predicted’ the observed percept better, while taking account of the inaccuracies of the perceptual system and what is known about how the environment should behave.

The percepts do not have to, and do not, correspond exactly to the state variables nor their representation. Instead, the state variables are related to the perceptual variables via various transformations (see equations (2.6)). Distance to the leading vehicle *d* is related to the leading vehicle's angular projection *ϕ*. The distance and speed relative to the leading vehicle *r* determine the angular expansion rate *v*_*ϕ*_, or ‘looming’. Own movement in a textured environment produces a visual movement pattern, or optic flow, whose magnitude *F* is related to the landspeed *v* [56].^4^

The percepts are assumed to be noisy, and estimates of the noises' distributions are assumed to be available. These transformations are used to predict what percept values should be observed by a given environment state configuration and the assumed noise distributions are used to weigh how much the state distribution should be adjusted based on accuracy of these predictions. This adjustment is done by optimally balancing the knowledge of the environment's behaviour (i.e. the predicted state) with the observed percepts using the Bayes theorem.

**Figure 3.**
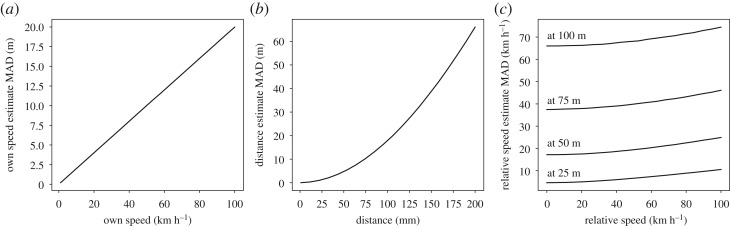
Single sample estimation errors for the state variables with the perception model (see equations (2.6) for the model and table 1 for the parameters). The error distributions are asymmetric and the error is given as median absolute deviation (MAD) of million simulated percepts. Own speed perception is modelled as a logarithmic transformation, so the error increases linearly with the speed (*a*). Relative distance to the leading vehicle is estimated from angular size and its error increases superlinearly (*b*). Relative speed is estimated as distance normalized angular expansion rate and its error (*c*) increases superlinearly with both the relative speed's magnitude and the distance relative to the leading vehicle (the four different lines). The actual estimation errors in the model are generally significantly lower because the model integrates samples over time and makes use of the relationships between the state variables (see main text).

All of the perceptual noise distributions are Gaussian and constant in the *percept space* (see equations (2.7)). Importantly, this means that the noise from the state variable perspective is not constant, but depends on the (estimated) environment state (figure 3). For example, because the optic flow percept *F* is assumed to be logarithmic with regard to the driver's speed, the estimate of the driver's own speed deteriorates in its precision as a function of its magnitude. Similarly, as the distance estimate is derived from the noisy angular percept, its precision deteriorates as the distance gets longer. Furthermore, this is compounded for the relative speed estimate, which is estimated from both estimated distance and perceived angular expansion, which both deteriorate as a function of distance.

**Table 1.** Parameters of the model and values for fixed parameters. Parameter values marked with * are estimated from data.

parameter	symbol	value
acceleration uncertainty threshold	$\sigma_{\hat{a}}^{⋆}$	*m s^−2^
flow noise standard deviation	*σ*_F_	0.3
angular width noise standard deviation	*σ*_*ϕ*_	0.3°
angular expansion noise standard deviation	*σ*_*v*_*ϕ*__	0.3° s^−1^
acceleration efference copy noise coefficient	*λ*_*a*_	0.1
expected leader acceleration standard deviation	$\sigma_{{\hat{a}}_{L}}$	4 m s^−2^
desired speed	$v_{\max}$	80 km h^−1^
desired time headway	*T*	*s
maximum desired acceleration	$a_{\max}$	* m s^−2^
maximum desired deceleration	$b_{\max}$	$a_{\max}/0.6$
acceleration exponent	*δ*	4
jam distance	*s*_0_	2 m
jam distance	*s*_1_	0 m

It should be stressed that the precision of the estimate at a given time instant is not determined solely by the percepts available and the model's estimation error is generally lower than that of ‘single percepts’ illustrated in figure 3. As the state estimation mechanism embodies knowledge about the relations between the state variables, it can produce ‘cognitive estimates’ even without direct perception: for example, an ‘additional’ relative speed estimate is produced just by observing changes in distance. In fact, especially the relative speed estimate is dominated by these ‘indirect’ estimates except at very close distances due to the quite rapid deterioration with increasing distance of ‘direct’ estimates from the angular expansion.

Formally, the driver's perception includes optic flow magnitude *F*, angular width of leading vehicle *ϕ* and rate of change of the angular width *v*_*ϕ*_. Perceptual variables are related to the environment using following transformations:

2.6 $$F^{⋆}(v)=\log(v)\;\phi^{⋆}(d)=2\tan^{-1}\frac{u}{2(d+d_{0})}\;v_{\phi }^{⋆}(d,r)=-\frac{4ur}{4{\left(d+d_{0}\right)}^{2}+u^{2}},$$

where *u* is leading vehicle width and *d*_0_ is the driver's distance from their vehicle's front bumper, which are both assumed to be known exactly by the driver. The driver further assumes its percepts are corrupted by Gaussian noise:

2.7 $$\hat{F}∼N(F,\sigma_{F}^{2})\;\hat{\phi }∼N(\phi ,\sigma_{\phi }^{2})\;{\hat{v}}_{\phi }∼N(v_{\phi },\sigma_{v_{\phi }}^{2}).$$

The transformations and noise distributions are used to derive a likelihood function for an observation given the predicted state, which includes all the percepts when the view is not occluded (*O*[*t*] = 0, see the next section) and only the optic flow otherwise (*O*[*t*] = 1):

2.8 $$\begin{array}{rl} \;f((F,\phi ,v_{\phi })|(v,d,r),O[t]) & =f_{N}{\left(F;F^{⋆}(v),\sigma_{F}^{2}\right)}^{1-O[t]}\\ & \;\times f_{N}{\left(\phi ;\phi^{⋆}(d),\sigma_{\phi }^{2}\right)}^{1-O[t]}\\ & \;\times f_{N}(v_{\phi };v_{\phi }^{⋆}(d,r),\sigma_{v_{\phi }}^{2}). \end{array}$$

The posterior density $\hat{S}[t]$ is formed from likelihood of the measured observation ***z***′[*t*] = (*F*′[*t*], *ϕ*′[*t*], *v*_*ϕ*_′[*t*]) and the predicted state estimate ${\hat{S}}^{\prime }[t]$ using the Bayes theorem:

2.9 $$f(\hat{S}[t]=s)\propto f(z^{\prime }[t]|s,O[t])f({\hat{S}}^{\prime }[t]=s).$$

The resulting density $f(\hat{S}[t]=s[t])$ does not have a closed-form solution and is approximated using a particle approximation scheme described in §3.3.2.

### 2.3. Acceleration distribution, uncertainty and attention

As illustrated in figure 1, the state estimate is used to continuously choose the appropriate action. The primary action in the CF task is the acceleration, and we model the acceleration choice using a traditional deterministic CF model of the form *a*(*t*) = *f*(*v*(*t*), *d*(*t*), *r*(*t*)), where *f* is the CF model that outputs an acceleration value for a given state. As the state estimate is a distribution of states, applying the model produces a *distribution of accelerations*. To get the required single acceleration value to output to the environment, we use the expected value of the distribution, although other central tendencies could be equally plausible. For the acceleration model, we use the Intelligent Driver Model (IDM) [57], although any other model of the aforementioned form could be used.

In the model, attention allocation is simulated using the self-paced visual occlusion setting, where the driver's view of the leading vehicle is blocked by an occlusion, which can be removed for a short period of time *t*_*G*_ (300 ms in the model and experiments) with the press of a button. This is taken to indicate that the driver requires additional perceptual information because of uncertainty cumulating over the occluded period [28,29,32].

The uncertainty in the acceleration distribution is used to control the attention. In other words, we propose that the requirement for visual samples is driven by *action uncertainty*, i.e. uncertainty in what is the proper acceleration for the situation, not perceptual or state uncertainty *per se*. We operationally define this as the standard deviation of the acceleration distribution. Visual sampling is modelled as removing the occlusion if the uncertainty rises above a threshold value (the individual's *uncertainty threshold*). Again, other uncertainty measures could be used, and in reality the sampling process in humans is likely stochastic itself, and dependent on the subtasks at hand, as was proposed by Johnson *et al.* [54]. (Of course, a more natural form of visual sampling would be to model gaze behaviour instead of the proxy of the occlusion—but this would require oculomotor control on the motor side, and the quality and quantity of peripherally available visual information to be explicitly modelled as well).

Formally, the acceleration distribution $\hat{a}[t]$ is formed by transforming the state estimate via the acceleration rule:

2.10 $$\hat{a}[t]=f_{\mathrm{IDM}}(\hat{v}[t],\hat{d}[t],\hat{r}),$$

and the acceleration output is the expected value of this distribution $\bar{a}[t]=E(\hat{a}[t])$ and the acceleration uncertainty is the standard deviation $\sigma_{\hat{a}}[t]=\sqrt{E(\hat{a}{\left[t\right]}^{2})-E{\left(\hat{a}[t]\right)}^{2}}$. If acceleration uncertainty is greater than the uncertainty threshold $\sigma_{\hat{a}}^{⋆}$ and the view is occluded, the driver removes (‘lifts’, *L*[*t*] at time *t*) the occlusion *O*, so that the view is unoccluded for the next *t*_*G*_ seconds:

2.11 $$L[t]=\left\{ \begin{array}{cc} 1 & \text{if }\sigma_{\hat{a}}[t]>\sigma_{\hat{a}}^{⋆}\wedge O[t]=1\\ 0 & \text{otherwise} \end{array}\right.$$

and

2.12 $$O[t]=\left\{ \begin{array}{cc} 0 & \text{if }\exists t^{\prime }\text{s.t. }t-t^{\prime }<t_{G}\wedge L[t^{\prime }]=1\wedge t\neq t^{\prime }\\ 1 & \text{otherwise.} \end{array}\right.$$

### 2.4. Empirical evaluation

Given a leading vehicle trajectory, the model, when parametrized, can produce a longitudinal trajectory the driver's vehicle takes along with time instances when the driver lifts the occluder. Thus, we are able to simulate the very same quantities that can be measured from human drivers in the experiment (see below).

A complicating factor for empirical testing is that, even with numerous simplifying assumptions, the model requires 13 driver specific parameters, of which seven are to parametrize the classical CF model. All of these parameters are internal to the driver and related to internal processes, and thus are not (directly) observable. However, thanks to the rich literature of both CF modelling and psychophysics, we were able to use reasonable fixed estimates for 10 of the parameters and model driver-specific variation with only three experimentally calibrated parameters.

## 3. Methods

### 3.1. Experimental methods

Two experiments were run, where longitudinal control and visual sampling were measured in a simple CF task. The same task was performed in a real instrumented vehicle, and in a simulation in immersive VR. For experimental control, the real car experiment was conducted on a test track, with the participant following a lead vehicle driven by an experimenter according to a pre-designed protocol. Visual sampling was measured by using the self-paced visual occlusion technique [28,29,32].

#### 3.1.1. Participants

A convenience sample of 40 subjects (22 M, 18 F; age mean 34 years, s.d. 10 years, range 22–59 years) were recruited through University of Helsinki and Aalto University mailing lists and personal contacts. The 40 subjects were selected from a pool of 64 interested individuals to approximately stratify sex, age and driving experience. The participants had held a driving license for an average of 15 years (s.d. 10 years) and had average self-reported kilometrage of 120 000 km (s.d. 150 000 km). The participants were required to have had a driving license for at least 5 years or have accumulated at least 30 000 km of driving.

The same participants conducted both the real and the simulated driving experiments. All participants conducted the real vehicle experiment successfully. Three participants did not complete the VR experiment due to discomfort.

All participants reported normal vision, and none reported strabismus or neurological diseases or medication that could affect their eye movement behaviour.

The participants were compensated for their time and effort by a reward of 100 euros (before taxes).

#### 3.1.2. Driving simulator

The driving simulator software was developed in-house and is available under an open source licence (https://github.com/samtuhka/webtrajsim/tree/vrsim). The experiment was run in a fixed base simulator set-up, comprising an HTC Vive VR headset (model OPJT100), a desktop computer (Corsair Anne Bonny with Windows 10 OS, Nvidia's GTX 1080 GPU, Intel's i7 7700k CPU and 32 GB of RAM), a distance-adjustable gaming chair (Playseat Evolution Alcantara, Playseats BV, The Netherlands), and a steering wheel and pedals game controller (Logitech G920 Driving Force, Logitech, Fremont, CA).

The VR headset had an approximate field of view of 100°, resolution of 1080 × 1200 per eye and a refresh rate of 90 Hz. The subjects' eye movements were recorded with Pupil Labs HTC Vive Binocular Add-on (Pupil Labs UG haftungsbeschränkt, Berlin, Germany).

The simulated vehicle dynamics parameters were decided by informal pilot testing to give a comfortable compromise of good controllability but not overly nervous responses.

#### 3.1.3. Instrumented vehicles

The participant-driven vehicle was a Toyota Corolla 1.6 compact sedan (MY 2007) with a manual transmission. The car followed was an instrumented Toyota Land Cruiser (M1G, 2007). The participant-driven car was instrumented for measuring driver behaviour and physiology and equipped with a brake and clutch pedal in the passenger footwell, so that the experimenter with a full view of the track at all times would be able to intervene if necessary.

The occluder used in the occluded CF task was custom-made IG Smart Glass that cleared when electricity was run through it (Switchable Toughened Glass 6 mm, Frosted White/Clear, Pro Display, Intelligent Glass UK). The occluder was connected to and controlled by a computer that registered the press of a control button on the steering wheel in such a way that the glass would clear for about 300 ms when the press-button was pushed, remaining opaque for the rest of the time (see electronic supplementary material, videos).

The driver's perspective of the scene was recorded, along with eye movements, using the scene camera of a Pupil Labs Binocular 120 Hz head-mounted eye tracker (Pupil Labs UG haftungsbeschränkt, Berlin, Germany). A custom-built headband was used to secure the headset more firmly.

GPS data were gathered via a mobile phone (Samsung Galaxy II GT-I9100 with Android version 4.0.4) placed at the centre of the control panel. CAN-bus data were collected for vehicle speed and accelerometer information. A laser scanner (IBEO Lux) mounted at the vehicle's front was used to measure distance from targets ahead. A Web camera (Logitech HD Pro Webcam C920) on the dashboard recorded the scene ahead of the driver. All data sources were collected to a laptop (Lenovo Legion Y520-15IKBN with Debian Linux operating system) and the data streams timestamped with unix timestamp, using the in-house developed Trusas data collection system available under an open source licence (https://github.com/jampekka/trusas-corolla).

#### 3.1.4. Location and track

Both experiments were conducted at the Helsinki-Malmi Airport (N60.2514°, E25.0513°), which has been decommissioned for commercial flights. An unused runway was used for the real car experiment, and a dedicated room inside the terminal building was set up as a pop-up laboratory for the driving simulator.

**Figure 4.**
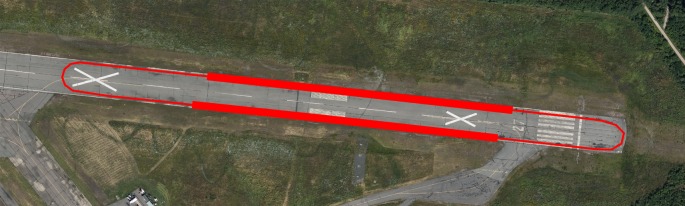
Aerial image of the airport runway used for the real car experiment. The red line shows the route driven continuously. The thickened parts indicate the parts of the route used for analysis. Aerial image copyright City of Helsinki, CC-BY-4.0.

The available runway had a length of approximately 500 m which was continuously driven back and forth, turning around at each end (figure 4). The turns and transitional phases on the straights before and after them were omitted from the analysis in order to get similar behaviour that would occur on a long straight road.

#### 3.1.5. Procedure

Two participants always began the experiment at the same time, and the real vehicle and VR experiments were conducted in parallel. Upon arrival, each participant was randomly assigned either a simulator first, real car second or a real car first, simulator second protocol by tossing a coin.

Participants filled a background information and informed consent form, and then proceeded to the driving tasks. After completion of the first one of the tasks, which took about 45 min, a short rest period was provided before taking the next part of the experiment. Altogether, including the breaks, the experiment took about 3 h.

**Figure 5.**
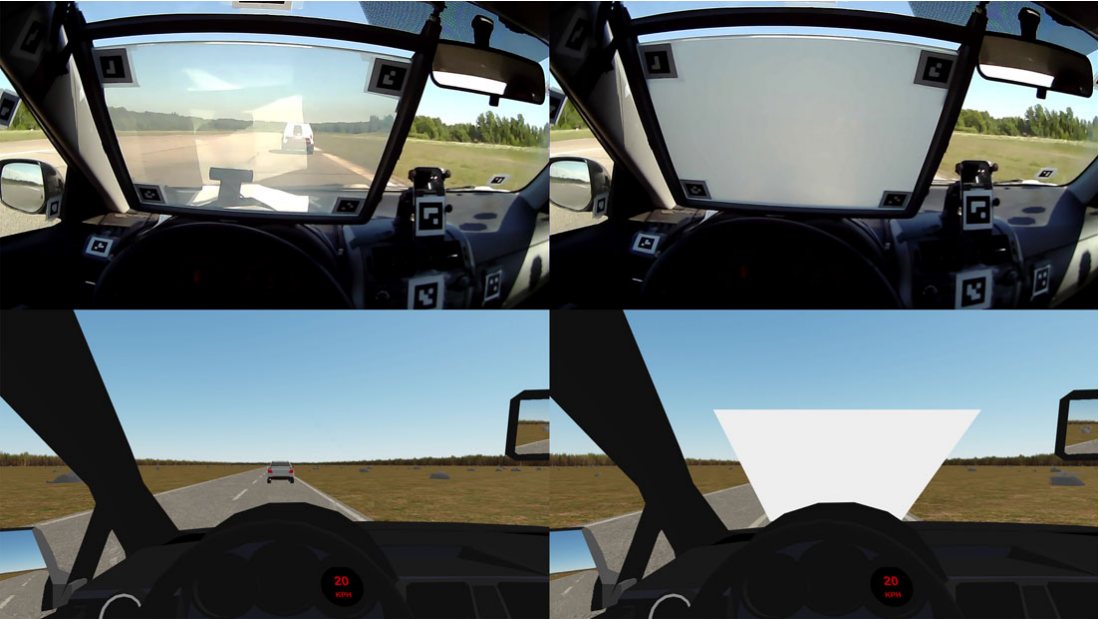
Participant view in the real car (top) and VR simulator (bottom) CF experiments with the occluder off (left) and on (right).

In the occluded CF task, a visual occlusion completely masked the driver's view of the leading vehicle (see figure 5 and electronic supplementary material, videos). In the VR experiment, this was a light grey rectangle displayed on the windshield; in the real car experiment the occlusion device described above was used. The participant could remove the occlusion by pressing a paddle shift lever in the steering wheel (VR) or a button on the steering wheel (track). This permitted a 300 ms ‘glance’, after which the occlusion returned for an indeterminate occlusion duration until the participant would request the next glance. The participants were instructed to ‘follow the car in front like you would in a crowded highway, but taking as few glances as possible’. No speed limits were given and overtaking and collisions were strictly prohibited.

In the real car experiment, participants first drove to the test site. After eye tracker calibration (data not used in this article), instructions were given to the participants. They then made a short test run to get used to the car and the track, and using the occluder with the push-button. After that, occluded and unoccluded versions of the task were randomly driven in 5 min trials—both versions four times (unoccluded version's data not used in this article). The experimenter (J.P.) monitored the experiment from the front passenger seat and was in position to intervene or stop the car in case the participant was at risk of hitting the car in front. The experimenter did not need to intervene at any time during the experiment.

In the VR experiment, the participants first had the eye tracker calibrated (eyetracking data not used in this article). Instructions were presented in text within the VR environment. The participants familiarized themselves with the controls via practice tasks involving approach to a stationary vehicle, speed control and lane changing. The three experimental tasks were first each performed once in order (CF, CF with occlusion and finally lane changing in traffic). After this, the participants performed several trials of each of the three main tasks in randomized order. There were three 300 s trials of the lane changing task and two trials of both the occluded and unoccluded CF tasks (each trial taking on average 225 s). The lane changing and unoccluded CF tasks are not analysed in this article.

In both experiments, the leading vehicle drove with a randomized speed profile, where a target speed was randomly chosen every 20–30 s from a set of three different speeds (20, 40 and 60 km h^−1^). The real car target was chosen randomly without any stratification and the experimenter attempted to accelerate/decelerate to the target speed by approximately 2 m s^−2^. The leading car dropped speed to take the turns at the ends comfortably and waited for the participant driven car to catch up after each turn. Each real car trial lasted for 300 s. For the VR simulator each target speed was driven three times during a single trial and the leading vehicle accelerated or decelerated to this speed by approximately 2 m s^−2^. The VR trial ended when the leading vehicle had completed each speed segment (taking on average 225 s) or if the subject collided with the leading vehicle.

### 3.2. Data preprocessing and analysis

The data analysis was done using custom Python scripts which, along with the data, are available under an open source licence at https://gitlab.com/mulsimco/follow17.

To estimate the distance to the leading vehicle in the real car experiment, the leading car was semiautomatically detected from the LIDAR produced objects. Speed was recorded from the car's CAN-bus reported wheel speeds. The acceleration was estimated as the time difference of Gaussian filtered (s.d. 1 s) wheel speed signal. Track position was estimated by projecting the GPS location to the estimated track polyline. An approximate ‘steady-state’ speed area of the track (figure 4) was defined based on timeseries of the drivers' speeds and data outside this area were omitted from analysis.

The VR simulator's physics model was logged at every frame, which gives an accurate position signal for both cars. Speeds and accelerations were computed from these position signals by taking first and second differences. Relative distance was computed as the difference of the vehicles' positions with the simulated vehicle's length subtracted.

For both experiments, the occlusion duration was calculated as the interval between successive button/lever presses, subtracting the unoccluded duration of 300 ms.

### 3.3. Modelling methods

#### 3.3.1. Parameter estimation

Estimating driver-specific CF model parameters from measured trajectories is known to be a somewhat challenging problem [58] which is exacerbated by challenges in optimizing stochastic systems. To avoid these issues, especially local minima and overfitting to compensate for model error, we make use of the controlled nature of the leading vehicle profile in our experiment. As the leading vehicle behaviour was statistically the same for each trial, we can produce a ‘virtual dataset’ of differently parametrized drivers and find a parametrization that on aggregate measurement level produces similar behaviour to each subject.

To simplify the parameter space, we fix all but three parameters to reasonable values based on the literature and our experimental set-up (table 1).

For the IDM [57], which we use as the acceleration function, the maximum acceleration $a_{\max}$ and desired time headway *T* are estimated for each driver from the data. Maximum desired deceleration $b_{\max}$ is assumed to be systematically related to the maximum acceleration as $b_{\max}=a_{\max}/0.6$ (based on the results of [59]) and target speed $v_{\max}$ is set to 80 km h^−1^, which is the Finnish speed limit of the type of road that was simulated. The rest of the parameters are set to the values presented in [57].

For the state estimation and attention model, the uncertainty threshold parameter $\sigma_{\hat{a}}^{⋆}$ was estimated from data for each participant, all others were fixed. The perceptual noise parametrization, *σ*_*F*_ = 0.3, *σ*_*ϕ*_ = 0.3, *σ*_*v*_*ϕ*__ = 0.3, was selected to produce approximately similar accuracy for distance, speed and relative speed as proposed in [60]. The expected leader acceleration standard deviation was set to the value of $\sigma_{{\hat{a}}_{L}}=4.0\;\text{m}\;{\text{s}}^{-2}$, which, on the timescale of few seconds, produces speed trajectories with statistically similar speed variation to those used by the leading vehicle.

The remaining free parameters, desired time headway *T*, maximum acceleration $a_{\max}$ and uncertainty threshold $\sigma_{\hat{a}}^{⋆}$, were estimated for each participant by finding parametrization that best matches the aggregate level performance of the subject using *k*-nearest neighbour regression. This was done by simulating 150 000 trials of the model performing the same driving task as the subjects did in the VR experiment (see §3.1.5) with random *T*, $a_{\max}$, and $\sigma_{\hat{a}}^{⋆}$ sampled from U(0.1 s,15 s), $U(0.1\;\text{m}\;{\text{s}}^{-2},8.0\;\text{m}\;{\text{s}}^{-2})$, $U(0.1\;\text{m}\;{\text{s}}^{-2},8.0\;\text{m}\;{\text{s}}^{-2})$, respectively, where U is the uniform distribution. Median time headway, median occlusion duration and 99th percentile of acceleration were computed for each of the simulated trials. For each subject, five closest matching simulated trials based on these features were selected and the average of the parameters generating these five trials were used as the estimated parametrization of the subject.

#### 3.3.2. Model implementation

The model is implemented as a ‘bootstrap filter’-type particle filter [61], meaning that we use the state transition model (equations (2.1)–(2.5)) as the proposal distribution. Simulations are run with timestep duration Δ*t* = 0.1 s, with *N* = 512 particles and with systematic resampling for each step. The percepts are corrupted with pseudorandom noise following the distributions assumed in the perception model (equations (2.7))

For this article's simulations, we use the following initial distribution: $(\hat{v},\hat{d},\hat{r})∼(v[1],U(5\;\text{m},200\;\text{m}),$ $U(20\;\text{km}\;{\text{h}}^{-1},60\;\text{km}\;{\text{h}}^{-1}))$, where U is the uniform distribution and *v*_0_ is the true initial own speed.

The desired acceleration, which is also the actuated acceleration output to the environment, is the weighted mean of the particle accelerations: $\bar{a}[i]=\sum_{k=1}^{N}w_{k}[i]{\hat{a}}_{k}[i]$, where the subscript *k* refers to *k*th of the *N* particles. If the corresponding standard deviation is higher than the uncertainty threshold $\sigma_{\hat{a}}^{⋆}$ the occluder is removed for the next 300 ms.

The model implementation in C++ with Python bindings is available at https://gitlab.com/mulsimco/cfmodels.

## 4. Results and discussion

This section will first replicate the results of our earlier experiment [28] regarding relationship between time headway and occlusion duration and compare the behaviour between the real car and VR simulator experiments. These are then followed by an analysis of the proposed model's behaviour and how it relates to the experimental results.

### 4.1. Experimental results

**Figure 6.**
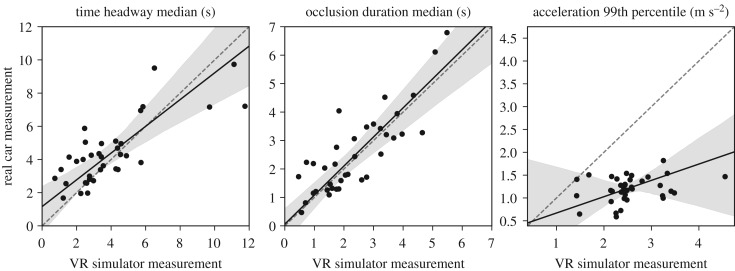
Comparison of per-subject (*N* = 37) aggregate measures in the VR and real car experiments. Dots indicate subject median, dashed line is one-to-one relationship, black line shows Passing–Bablok regression result and the grey area its 95% confidence interval. Time headways (Pearson *ρ* = 0.78) and occlusion durations (*ρ* = 0.83) correspond quite closely, with almost one-to-one linear relationships. Correlation of 99th percentile of accelerations is low to non-existent (*ρ* = 0.29, *p* = 0.09) and VR simulator results in about twice larger acceleration peaks.

Comparisons of per-subject aggregate measures in the VR and real car experiments are illustrated in figure 6. Real car time headways and occlusion durations are predicted quite well by the VR simulator data, indicating good external validity of the simulator with regard to these variables. Between subject variation in acceleration behaviour on the other hand is not practically correlated at all, and about twice the real car acceleration range is produced in the simulator experiment. This is likely a combination of much more responsive dynamics of the simulated car and lack of somatosensory acceleration and jerk information, exacerbated by relatively subdued leading vehicle accelerations and speeds.

**Figure 7.**
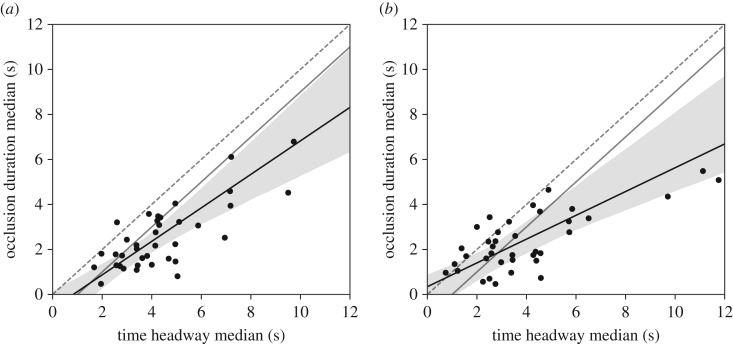
Between-subjects relationships of median time headway and occlusion duration in the (*a*) real car (*N* = 40) and (*b*) simulator (*N* = 37) experiments. Dots indicate per-subject medians, dashed line is one-to-one, black line is Passing–Bablok regression line and grey area its 95% confidence interval. Grey solid line is our previous proposal of $\hat{o}\approx {\hat{T}}_{D}-1$ [28]. The relationship is quite strong and relatively well explained by a linear relationship (real car Pearson *ρ* = 0.76, VR simulator *ρ* = 0.73). Our previous proposal is reasonably close to the current data, although there is a statistically significant departure from its exact parameters in both settings.

Our earlier result that between-subjects level time headway and occlusion duration have a strong correspondence [28] is replicated by both the real car and simulator experiment (figure 7). There is a statistically significant departure from the exact parametrization $\hat{o}\approx {\hat{T}}_{d}-1$, although we still regard it as a reasonable approximation.

We also find that the time headway and occlusion duration correlate at within-subject level. To remove possible spurious correlation due to changes in the time headway tendency, robust linear trends of both time headway and occlusion duration were removed from each trial as in our previous study [28]. After the detrending, a positive Spearman correlation is observed for 28 of 40 participants in the real car experiment (Binomial test *p* = 0.02) and 31 of 37 in the VR simulator (*p* = 0.00004). The correlations (real car grand median 0.15, VR grand median 0.19) are however considerably lower and less consistent than in our previous simulator experiment, where all subjects had a positive correlation with grand median of 0.57. This is likely at least partly due to the more subdued speeds and accelerations of the leading vehicle (mandated by safety and practicalities of the real car experiment) leading to less variation in the within-subject time headways. Owing to the low correlations, in contrast to the previous study, we do not estimate slopes for the relationships.

### 4.2. Modelling and simulation results

**Figure 8.**
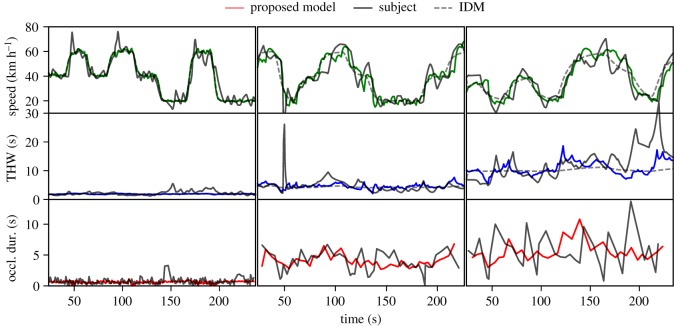
Time-series samples of human and model produced trajectories and visual occlusions. The three columns represent a VR simulator trial of three different human drivers and models calibrated to their data. Dark solid lines represent the human driver, dashed solid line represents the classical IDM with continuous input and the coloured lines represent proposed model's driven sample trajectories. The rows are from top to bottom: speeds, time headways and occlusion durations. The plotted occlusion duration is the duration of the occlusion *following* the glance at the *x*-axis indicated time, and the line is formed by linearly interpolating this value between adjacent glance instants. Video reconstructions of the human and model drives are available in the electronic supplementary material.

On the general level, the model can successfully drive our CF scenarios with intermittent and noisy input, and the parameter estimation procedure (see §3.3.1) captures aggregate level between-subjects differences. Figure 8 shows time-series level comparison of human and model performance. The overall level of speed, time headway and occlusion duration is replicated quite well by the model, but this is to be expected as the parameter estimation procedure optimizes the model to match per-subject aggregates (medians) of time headway and occlusion duration and 99th percentile of acceleration.

On the more detailed level, the human performance has a significant amount of short-timescale variation in the speed, distance and visual sampling behaviour. This is somewhat oscillatory in nature and likely has a considerable stochastic component, i.e. having the same participant drive the identical scenario multiple times would result in somewhat different trajectories. The variation in the model produced trajectories shows similar behaviour, although the model shows somewhat less short-timescale variation than the human drivers. This is likely at least partly due to the model not including acceleration noise, which is known to add such short-timescale variation like seen in the human trajectories [17].

Considering that the model is stochastic and particle-approximated with noisy and highly intermittent sampling, it is surprising how rarely it collides with the leading vehicle. Based on 300 simulated trials for each subject's estimated parameters, on average 1.1% of real car trials and 0.8% of VR simulator trials should end in a crash. The observed rates were 0% (95% CI 0–2.4%) in the real car and 3.3% (95% CI 0.9–8.4%) in the VR simulator.

**Figure 9.**
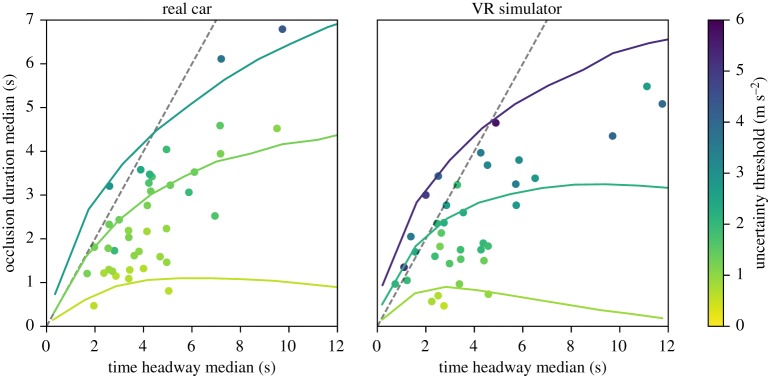
Model-predicted relationships of median time headway and occlusion duration at different uncertainty thresholds. The coloured lines represent the simulated relationship at estimated 5th, 50th and 95th percentile uncertainty thresholds of the subject population. Dots represents per-subject medians with colour indicating the subject's estimated uncertainty threshold. The dashed line is one-to-one.

On the between-subjects level a systematic relationship between median time headway and median occlusion duration emerges from model-driven trials when holding all but the desired time headway parameter constant (figure 9). The relationship is notably nonlinear, but it matches relatively well to the experimentally observed values. Also the model gives a tentative explanation that the between-subjects variation in the relationship may be largely due to different levels of accepted acceleration uncertainty, with the relationship also modulated by the desired acceleration levels. The simulator has significantly higher uncertainty thresholds, which is largely explained by higher desired accelerations.

The model also exhibits the shorter timescale within-subject relationship between detrended time headways and occlusion durations that was found in the current data as well as in our previous results [28]. With real car subject parametrizations the median Spearman correlation is 0.65 and VR simulator parametrizations 0.45.

### 4.3. Discussion of the results

The results clearly indicate that proposed model can ‘drive’ successfully with noisy and intermittent sampling of the environment. It can also capture aggregate level between-subjects variation time headway and visual sampling behaviour with only three estimated parameters. The previously found, and now replicated, correspondences between time headway and occlusion duration are found to emerge from the model, even though they are not explicitly included.

However, the question of how ‘well’ the model matches the human behaviour is not trivial to answer. The output of the model is stochastic, and thus straightforward ‘goodness-of-fit’ measures cannot be given at least with the current model fitting procedure. Which aggregate level measures to compare is somewhat arbitrary and for this reason we have restricted the analysis to the variables we used in previous work. Also it is quite clear that there is still systematic human behaviour not replicated by the model, especially the short-timescale variation is substantially smaller in the model produced trajectories. A clearly missing major component causing lack of variation is the control model, which should in the least include some sort of acceleration noise.

It is not clear how the experiment corresponds to real-world driving. Of note here are the quite long time headways: medians of subject medians were 4.1 s for the real car and 3.4 s for the VR simulator experiments, whereas the typical observed median time headway for the kind of road described in the instructions is well under 3 s [62]. In traffic engineering such long time headways are often associated with free flowing traffic, as opposed to CF where the leading vehicle limits the drivers speed choices [63]. However, free flow driving is likely not the reason for the long headways observed in our experiment: the leading vehicle speeds were as low as 20 km h^−1^, which should be clearly below free flow speed for even the most cautious driver, and high time headways were observed at all speeds. Furthermore, as the time headways correlate well with the occlusion durations, much of the abnormally long time headways is likely due to the subjects opting for significantly higher (visual) distraction levels than they would in normal traffic, which are compensated by higher time headways.

The crash-rate analysis should be taken mostly as an indication that the model is able to perform the task at somewhat similar level to humans, and the model crash rates are somewhat deflated because it can actuate decelerations that were not possible in the real car nor the VR experiment. The collision rates are clearly higher than they would be in normal traffic, but they most likely are for the human drivers also in this task, where the level of overt distraction is at least for some participants considerably higher than they would tolerate in real world with no extra safety precautions and experimental instruction, which is quite clear at least for the VR simulator based on the observed collisions. Although the experimenter did not have to intervene at any point in the current experiment, the experimenter's (J.P.'s) educated guess is that given enough repetitions a significant intervention rate, perhaps even around the model predicted 1%, would emerge.

## 5. General discussion

We have presented a CF model that, based on stochastic internal representation and a psychologically plausible perception system, successfully simultaneously handles longitudinal control and intermittent sampling with performance comparable to humans. The model can be calibrated to capture individual differences and exhibits adaptation of attention similar to what is empirically observed in human driving. With the internal representation, it can handle noisy input, drive extended periods based on only predicted dynamics of the environment and ‘know’ when input is needed.

The need for input in our model is driven by *action uncertainty*. The general idea that attention is driven by uncertainty is quite well established [29,64,65], and more recently computational attention sharing models using estimate of state uncertainty have been developed [54]. However, this earlier work has focused on uncertainty about the environment, which poses a crucial problem of state variable relevance—even in the quite simple case of CF. Namely, *which state variables* have to be known with *what certainty* in order to drive without perception? For example, it is quite clear that accurate estimate of relative speed is not very crucial if the distance to the leading vehicle is very high, or that accuracy demand for an overtaking car in a neighbouring lane depends greatly on whether the driver herself is about to switch lanes or not. Using the action uncertainty sidesteps such problems as the importance of different variables is implicitly included in the decision rule. On a more general level, it can also be taken as a reflection of the idea that the primary purpose of perception is to serve action, not necessarily to infer an accurate and veridical world model [66].

### 5.1. Implications for traffic psychology

The theoretical basis for studying the time headway–occlusion duration connection was based on the task-difficulty homeostasis of Fuller [27] and how it should be modelled in CF [28]. Previously we and others [13,14] have proposed that in the CF task the task demand is mostly determined by time headway and that drop in capability due to, for example, distraction will cause the driver to increase the time headway in order to keep the demand and capability in balance. Empirically, this seems to be supported; time headway is well *correlated* with task demand, assuming that the capability is connected to visual distraction and the homeostasis is in effect. However, in light of the current model, a more appropriate measure for task demand may be the action uncertainty. This is not in contradiction with the idea of time headway as an index of task demand, but our formulation proposes that time headway's correlation with the posited task demand *emerges* from the more complex underlying control of action uncertainty. Action uncertainty has also the practical benefit that it can be generalized to different and more complicated tasks than task specific measures like time headway.

It should be noted that the proposed model only handles ‘one half’ of the balancing of task demands and capability: increasing of capability by attending to the task in order to bring the demand caused by action uncertainty to a satisfactory level. The other half—decreasing action uncertainty by, for example, increasing the time headway in order to ‘free’ capability, e.g. to attend a secondary task—is not currently included. This latter mechanism is the one studied in the aforementioned CF models incorporating the task–capability homeostasis. These models could be quite straightforwardly integrated into our model's framework, as they are formulated as acceleration rules, but with an additional distraction level parameter.

As mentioned in the Introduction, the model incorporates many features of the ZRT [19]. Especially prominent influence is the central role of expectancies, or prediction of the immediate future, which are a prerequisite ‘for any success in driving performance’ [19, p. 188] according to the ZRT. In fact, our model in a sense uses *only* expectancies, in that perception, when available, is only used to ‘evaluate’ predicted environment states.^5^ The model is also ‘zero risk’ in that it does not compute or use any probabilities of adverse events, as is done in the so-called ‘risk control models’ [24–26] and in CF models based on expected utility [15,16].

On the other hand, the action selection process and uncertainty are not very prominent in the ZRT, and attention and vigilance are mostly discussed in relation to environment. The action uncertainty perspective could prove useful in developing the ZRT further, especially by producing a potential mechanism for the ‘risk monitor’, which has previously been somewhat difficult to reconcile with the theory's lead motif that risk estimation does not enter the control loop in routine driving. It should be noted that ZRT mainly discusses behaviour in somewhat higher level than the very short timescale speed control of the CF task, and what the current model is doing could be interpreted as performing the ‘routine driving’ which the higher level mechanisms, such as motives and behaviour adjustments due to risk monitor activation, modulate usually on longer timescales. Further model development should explicate such modulations in a computational form, and be combined with experimental settings where the risk monitor mechanisms should come into play more prominently.

An interesting development could be to integrate ideas from the utility maximization of risk control models into the higher level modulation. The ZRT is quite explicit in that subjective risk is a part of the driving process, but that it is used to adjust behaviour in response to risk monitor activation. Within the computational framework, this could be implemented so that whenever the risk monitor gets activated, due to, for example, prolonged action uncertainty, a risk computation would be conducted and the *parameters* of the control loop would be adjusted, e.g. desired (habituated) time headway could be increased in response to an unpredictable leading vehicle. This would alter the risk control mechanism so that risk is not continuously computed and reacted to, but rather risk would be associated with using a given control mechanism or its parametrization in a given situation.

### 5.2. The predictive processing perspective

With regard to unifying psychological and computational process models, we would consider particularly apposite the concepts deriving from the literature on prediction, sensory integration, stochastic internal models and uncertainty in them [64,65,67,68]. Relevance of these ideas for understanding various driving scenarios has been recently discussed by Engström *et al.* [69], and some of the ideas are incorporated in the recent computational modelling framework of Markkula *et al.* [70].

Our model incorporates some components that are quite central in this so called ‘predictive processing’ framework: it makes use of a predictive model, Bayesian sensory integration and models attention using uncertainty. Especially, the way our model combines the top-down dynamics model with bottom-up percepts by taking into account their respective estimation variances^6^ is essentially identical to how evidence is accumulated in the predictive processing framework.

However, the framework also has number of central concepts not included in our model. One of them is *hierarchical* predictive models, but this is mainly due to the present work being the first pass attempt. We concede that a hierarchical structure is likely necessary in order to integrate more complicated behaviour, such as lane changing, combined longitudinal and steering control in curve driving, and especially the unified treatment of gaze and locomotor control (see [71] for detailed discussion).

### 5.3. Intermittency in action versus control

Another interesting parallelism in our model and some recent models based on prediction errors [70,72,73] is the treatment of intermittency: our model uses intermittent perceptual sampling to drive continuous motor control, whereas these ‘prediction error models’ use continuous perceptual sampling to drive intermittent motor control. Specifically, the models propose that control is based on intermittently applied ‘motor primitives’ which—compared to simple continuous adjustment as in the present model—are relatively complex manoeuvres, akin to ‘precognitive responses’ discussed in some formulations [31,74].

These approaches need not be mutually contradictory, however, and the most realistic model might turn out to be one based on intermittent sampling driving intermittent control responses. The choice of a simple continuous control for the motor control was mainly driven by a desire to make the present model directly applicable to traditional agent-based microsimulation models, and to pinpoint the effects on traffic flow in the variation of attention. More complex motor control mechanisms are likely to be needed if our model is to be generalized to more varied control tasks, which again immediately arise when, for example, modelling vehicle dynamics, combined effects of speed control and steering or the coordination of gaze and locomotion.

That said, we would like to point out some more substantial differences between the present approach and the predictive intermittent control models that will need to be empirically or theoretically resolved in the future.

We emphasize the cognitive state estimate—the mapping from percepts to motor actions is not straightforwardly based on monitoring learned visual cues, but mediated by a state representation, which we envision as a system capable of supporting learning and the coordinated control of multiple simultaneous tasks. That is, we envision a much larger role for internal state representations in models aiming for the scope of full longitudinal control as opposed to only braking or lane keeping. More concretely, in our model, control is driven by the internal model of world state (‘expectancy’ in ZRT terms). In the predictive intermittent models control is driven by errors in outcome prediction—that is, differences between the state variable values predicted from the motor sequence efferent copy and the observed variable values. That is, in the Markkula *et al.* framework [70], a change in control is dependent on time-delayed feedback, it cannot be changed ‘mid-flight’ on the basis of internal predictions as such. At the moment, it is not known whether this limitation applies to human motor control.

However, overall at the conceptual level our model sits quite well in the framework of Markkula *et al.* [70], which is based on prediction of sensory inputs, efference copies of own actions and accumulation of evidence. Also the concept of attention allocation via action uncertainty should be quite straightforward to extend to the motor primitives type control, given a mapping between (estimated) environment states and appropriate motor primitives. Conversely, the kind of intermittent control envisioned in Markkula *et al.* may have uncertainty reducing effects which could be modelled in the present framework. Specifically, as motor primitives (synergies, motor programs, precognitive responses) produce ‘efference copies’ this should decrease uncertainty in the state estimates (increased task capability, reduced attentional demand for visual sampling).

On a deeper level, the strict differentiation between the state estimation and motor control may be a conceptual simplification that will need to be conceptually revised in the future. Again, the clear separation between the acceleration model and state estimation in the current formulation is largely to maintain direct comparability with the existing CF literature.

### 5.4. Limitations and future developments

Although we aim for psychological plausibility, we have had to make numerous idealizations to keep the parametrization and scope manageable. Perhaps the clearest idealization is our lack of a physiologically and physically plausible control model: the ‘simulated driver’ has a perfect and instantaneous control over the vehicle and can produce accelerations that are not physically possible. This is not, however, a limitation of the general approach; the model readily supports any acceleration model that is based on the ‘usual’ scene operationalization of speed, distance and relative speed and more physically plausible acceleration models (e.g. [72,73,75]) should be integrated in the future.

Using a ‘perfect’ control model does have the benefit that it is possible to pinpoint the deviation from traditional deterministic models to the introduction of the perceptual and attention sharing model. For example, the noise and intermittency of the perception lead to similar control effects to those that are often attributed to imperfections and intermittency in the acceleration control [17,72,73]. This is not to say that the models contradict each other; as discussed above, the current acceleration model lacks realism, which could be remedied by these more plausible models. Furthermore, imperfections in the acceleration lead to more variation in the true and estimated state, which increases attentional demands. The computational formulation also makes it possible to directly estimate, or at least quantitatively hypothesize, these effects.

Also, the perception model's relationships (see §2.2) are selected mostly for their compatibility with the usual CF model state formulation and for producing functionally similar estimates that are usually accepted in psychology. Specifically, when combined with the perceptual space noise, they approximately follow the Weber–Fechner Law in that the precision of the estimates of distance and speeds is strongly related to their magnitudes, which is a robust finding in psychophysics and includes distance judgements and visual speed estimates [56,76–79]. The exact forms of these relationships and mechanisms behind them are a contentious issue, and by no means our exact choices in the transformations reflect any scientific consensus.

Especially, the question of how relative speed is judged is an important topic in study of locomotion: the influential tau-theory shows that time to contact to an object can be estimated ‘directly’ by computing angular expansion rate relative to the angular size, or *τ*, and proposes this measure is used for guidance of action [80], but it has been shown that *τ* does not at least solely explain time-to-contact estimation [81,82]. However, our selection of ‘distance-normalized angular expansion’ is primarily motivated by it being more directly applicable to existing CF models. CF models operate on relative speed instead of time-to-collision, and thus *τ*-based perception model would have to include a time-to-collision to relative speed transformation.

In general, our assumption that transformations can be done accurately is clearly an idealization, and the decision of at which points of the transformation chain measurement noise is applied can affect the state estimates significantly. This means that the aforementioned separate study of subsystems, while likely necessary, is not sufficient for modelling full task performance.

The importance of active visual sampling [83–85], and its basis in reduction of (task-relevant) uncertainty, are pervasive ideas in understanding naturalistic task performance [86]. But although we boast about modelling a ‘naturalistic’ task, it has to be admitted that drivers rarely have their view totally blocked by an opaque piece of glass, and the drivers have to steer in addition to controlling speed, although based on previous experiments without steering [28], its effects on longitudinal control in a straight road are likely quite small. We maintain that the current setting is relatively representative of distracted driving on a long perfectly straight one-lane road, with perhaps the lack of peripheral vision and sampling with actual eye movements being the most glaring omissions, but acknowledge that generalizing the model beyond this very task needs additional work.

Peripheral vision could be modelled quite easily in the current formulation by changing the perceptual noise levels as a function of the target's visual eccentricity [87,88], and slightly less easily but more realistically by explicitly modelling the type of perceptual input available through the visual periphery according to current models [89]. However, generalization to tasks where the ‘gaze target’ is not *a priori* obvious, such as curve driving [90,91], causes complications for the action uncertainty mechanism: in order to lower uncertainty of the action, the model would have to know not just *when* but *where in the scene* to attend to get information about which action to take. This somewhat negates the convenience aspect of using the action uncertainty, but provides an interesting computational perspective on the inseparability of perception and control in natural behaviour. One possible approach would be to base the choice of the location attended on a predictive forward model of how different locations would affect (steering) control uncertainty.

Finally, although much of the background in our work comes out of traffic simulation, the current article does not evaluate the model's performance and effects in simulated traffic. This is partly a deliberate omission to keep the scope of the article manageable, but it is also unclear how well the results, even though driven with a real car, generalize to real traffic. The real car experiment was conducted on an empty track in an instrumented vehicle with a distraction simulated using visual occlusion, rather than in real traffic with natural secondary tasks. It may not accurately reflect the participants' true driving habits.

Also, in order to get variation in the data, the leader's speed is much more volatile than what would be representative of actual highway conditions. This may be partly behind the model's crash rate which—though remarkably low for such impoverished input and simplistic state representation—is likely higher than participants', and certainly much higher than in traffic. The fact that the model does produce collisions and mostly due to the highly plausible reason of inattention [92] could be used in the future to study safety effects of drivers' attention allocation mechanisms in single driver and traffic system level.

The ‘cognitive’ part of the model has only one subject-dependent variable, the uncertainty threshold. Although we required normal vision and reasonable driving experience from the participants, there is still clearly variation in, for example, perceptual accuracy, predicting ability and control skill that is not captured, and/or is being included in the other parameters, thus hindering their interpretation. To get better parameter estimates and to validate assumptions of the subsystems, tasks where different aspects, such as perception and control, are better separated could be studied.

## 6. Conclusion

Based on the concepts drawn from perceptual psychology, cognitive science, traffic engineering and computer science, and an experiment conducted in both immersive VR and a real instrumented car, we developed a computational model of driver visual attention and longitudinal control during a routine CF task. The model provides a possible mechanistic basis for the correlation of median time headway and occlusion durations across individuals, and also for the deviation from a simple linear relation. The attention mechanism is based on control of a cognitive internal estimate of *action uncertainty*, which is a conceptually novel proposal for attention allocation.

The model brings to bear probabilistic (Bayesian sensory integration) and predictive processing ideas from cognitive modelling, to develop a cognitively plausible driver model for CF scenarios. It potentially unifies a number of classical psychological hypotheses—such as driver sampling to maintain a sufficiently accurate ‘image’ studied in classical visual occlusion experiments [29,32] (cf. also [33,93]), the routine control of safety margins, and actions driven by an ‘expectancy’ of the future development of the situation [19,21]. The work also has the potential for harmonizing the psychological literature with the more rigorous but less psychologically plausible driver modelling in engineering.

## Supplementary Material

Sample trials of simulated and human driving

## Footnotes

1 We describe the model as a discrete time system in order to stay close to the implementation.

2 The magnitude-related noise is formalization of an assumption that the efference copy prediction follows similar magnitude-related precision as most other perceptual processes. Intuitively, it is assumed that outcomes of smaller control adjustments are predicted more accurately than of larger adjustments. See §5.4 for further discussion of magnitude-related precision.

3 The predicted state distribution ***S***′[*t*] corresponds to the predicted estimate *p*(***s***[*t*]|***z***[*t* − Δ*t*], …, ***z***[0]) in the usual Bayesian filtering notation, where ***z*** are observations and ***s***[*t*] are realizations of the random vector ***S***[*t*].

4 In reality, perception of even such a relatively simple scene takes account of a vast amount of cues. We have chosen this set of percepts for simplicity and relatively straightforward compatibility with the state estimate. See §5.4 for further discussion.

5 In fact, in the original schematic figure of the ZRT [19, fig. 21], perception affects action selection only through expectancy.

6 The predictive processing literature usually discusses this in terms of precision, which is the inverse of variance.

## Data accessibility

The data and analysis scripts are available online at https://doi.org/10.5281/zenodo.1341081 [94].
